# Corrigendum: Sequences flanking the core-binding site modulate glucocorticoid receptor structure and activity

**DOI:** 10.1038/ncomms13784

**Published:** 2016-11-22

**Authors:** Stefanie Schöne, Marcel Jurk, Mahdi Bagherpoor Helabad, Iris Dror, Isabelle Lebars, Bruno Kieffer, Petra Imhof, Remo Rohs, Martin Vingron, Morgane Thomas-Chollier, Sebastiaan H. Meijsing

Nature Communications 7: Article number: 12621; DOI: 10.1038/ncomms12621 (2016); Published: 09 01 2016; Updated: 11 22 2016

The financial support for this Article was not fully acknowledged. The Acknowledgements should have included the following:

M.B.H. and P.I. are grateful for computational resources provided by the North-German Supercomputing Alliance and the ZEDAT cluster Soroban of the Freie Universität Berlin.

