# Supplementary material for: Sequences flanking the core-binding site modulate glucocorticoid receptor structure and activity
Source: Nat Commun. 2016 Sep 1;7:12621. doi: 10.1038/ncomms12621 (PMC5025757; doi:10.1038/ncomms12621)
Supplement: Supplementary Information — Supplementary Figures 1-10 and Supplementary Tables 1-5. [file ncomms12621-s1.pdf]

Supplementary Figures and Tables

Supplementary Figures:

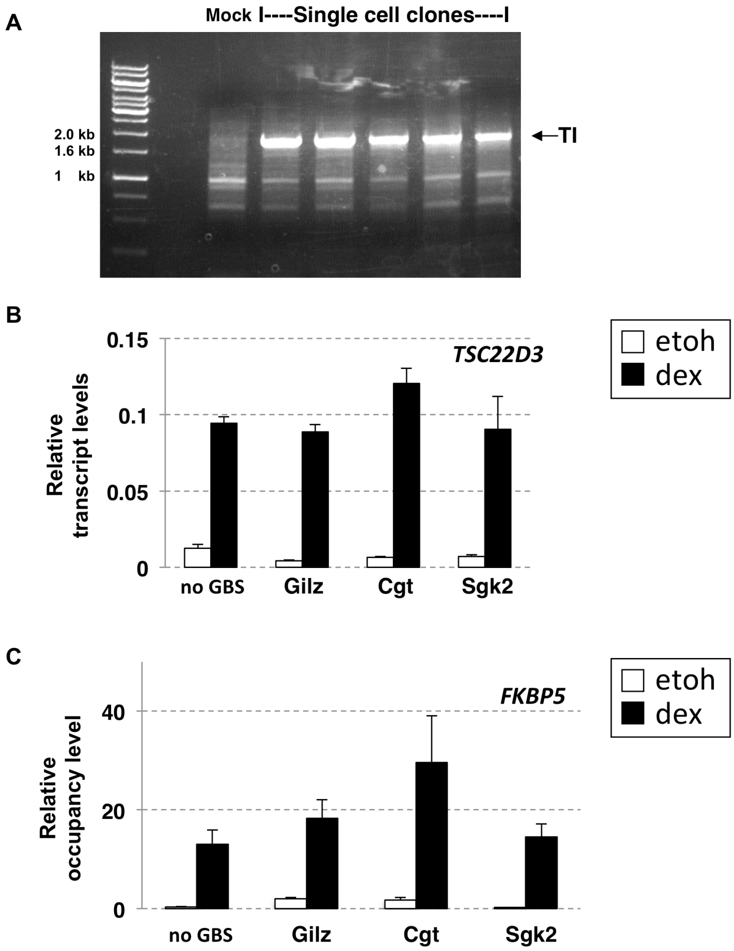

Supplementary Figure 1. Clonal lines show comparable regulation of endogenous target genes and binding to endogenous loci. (A) Correct integration at the *AAVS1* locus was assessed by PCR using primers that span the newly formed junction. (B) Transcriptional activation of the endogenous *TSC22D3* target gene was determined by real-time quantitative PCR (qPCR) for each of the clonal lines. Average relative transcript levels for three clonal lines per reporter for treated (1µM dexamethasone, 8h) and untreated (etoh vehicle) cells ±S.E.M. is shown. (C) GR occupancy at the endogenous *FKBP5* locus was determined by qPCR. Average occupancy for at least three clonal lines per reporter for treated (1µM dexamethasone, 1,5h) and untreated cells ± S.E.M. is shown.

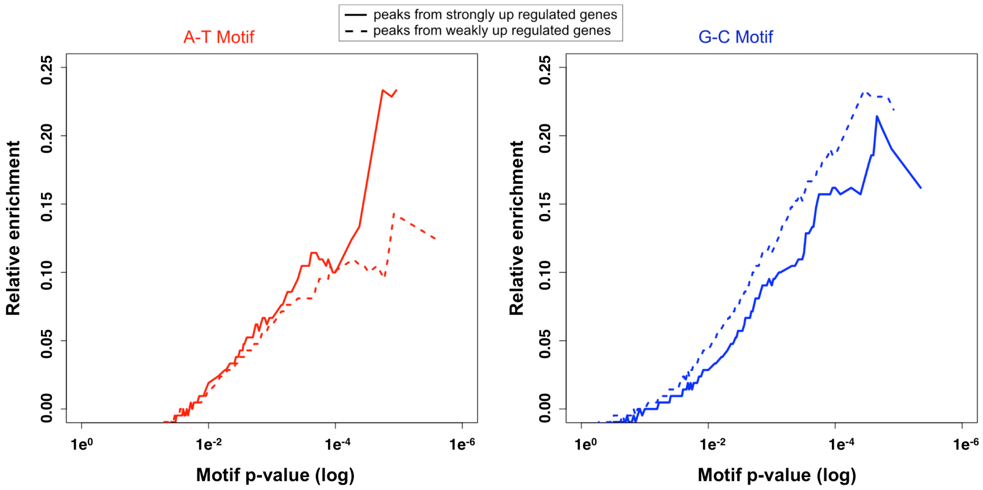

Supplementary Figure 2. Comparison of p-value distribution of A/T and G/C motifs. Plots showing relative enrichment of motif over motif p-value of A/T-flanked GBSs (left) and G/C-flanked GBS (right) for peaks associated with strongly upregulated genes (solid line) compared with peaks associated with weakly upregulated genes (dashed line).

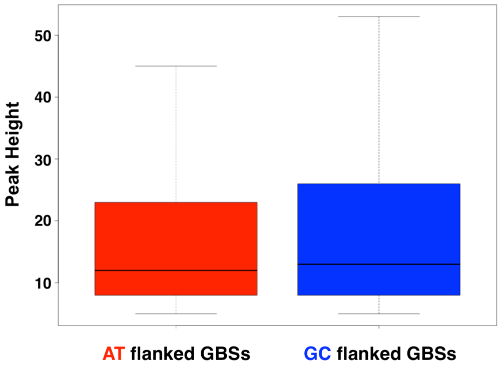

Supplementary Figure 3. Comparison of peak heights between ChIP-seq peaks harboring either A/T or G/C flanked motifs. Boxplots showing average peak-height for all peaks containing A/T flanked or G/C flanked GBS from GR ChIP-seq in U2OS cell lines. Center lines show the median, box limits indicate the 25th and 75th percentiles as determined by R software; whiskers extend 1.5 times the interquartile range from the 25th and 75th percentiles. Wilcoxon rank-sum test shows no significant difference in peak-height (p value=0.2208) between both groups.

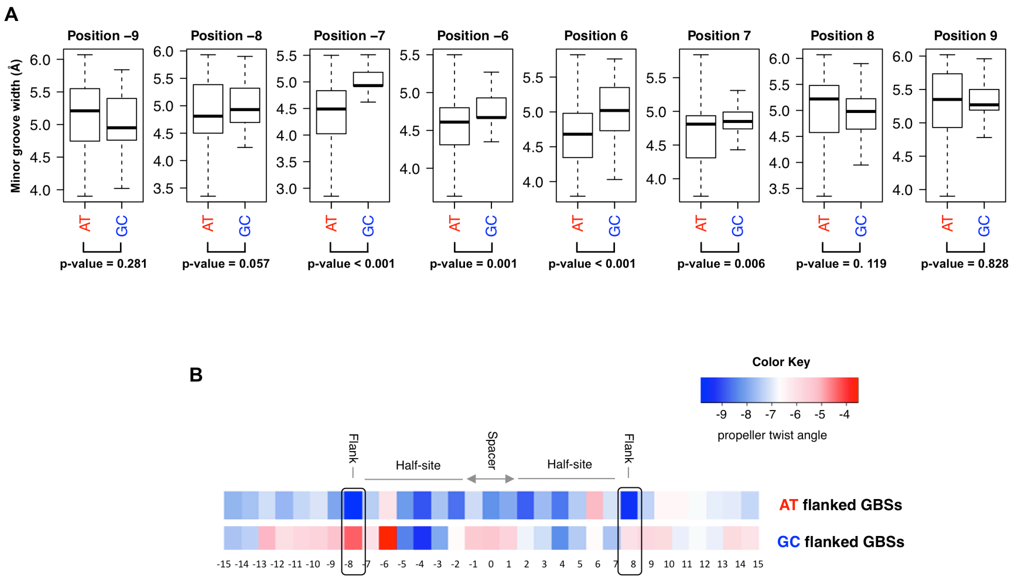

Supplementary Figure 4. Effect of flanking sites on DNA structure. (A) Minor groove width for select individual bases of the GBS for group of A/T-flanked GBSs associated with strong responder genes (83 GBS) and for group of G/C-flanked GBSs associated with weak responder genes (75 GBS). The boxes show the range between the 25th and 75th percentiles, the line within each box the median and the outer lines show the range between the 5th and 95th percentiles. p-values were calculated using two-sample, two-sided Wilcoxon tests. (B) Average propeller twist of individual bases from GBSs grouped into A/T-flanked GBS sfrom strongly up-regulated genes and G/C-flanked GBSs from weakly up-regulated genes (sequences as in Fig.4A).

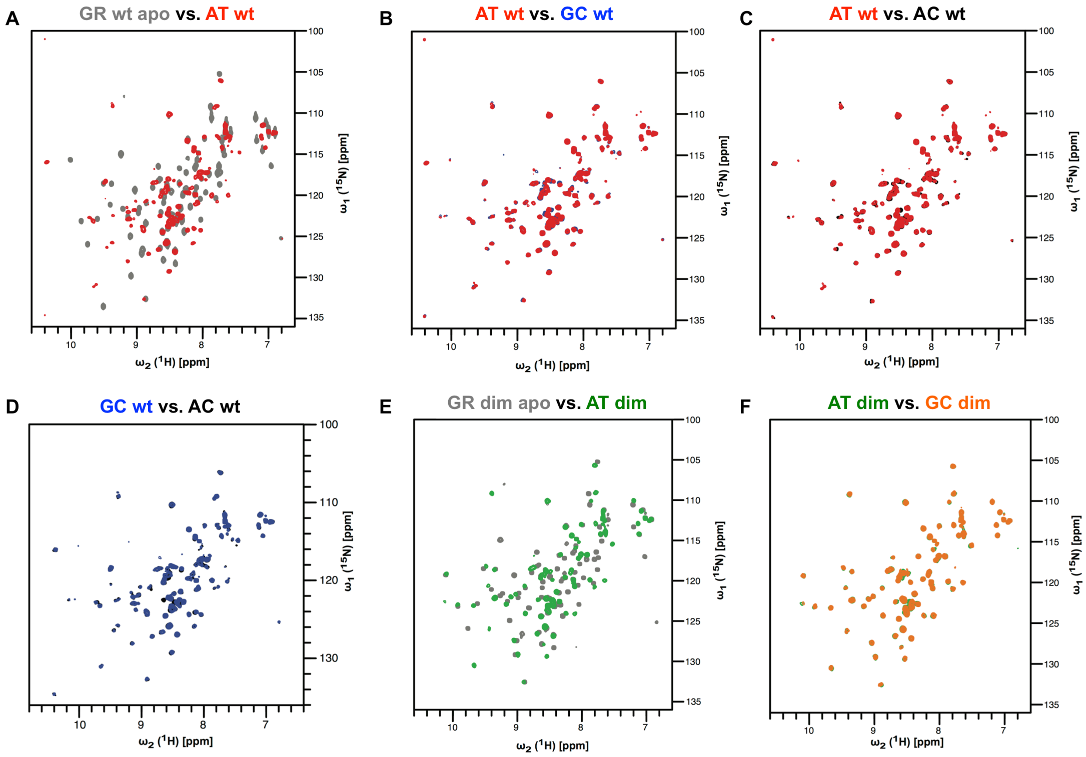

Supplementary Figure 5. ^{15}N-HSQC spectra overlay for various DNA:GR DBD complexes. (A) GR wild type DBD apo (grey) versus wt DBD:A-Cgt-T (red). (B) Wild type DBD A-Cgt-T (red) versus G-Cgt-C (blue). (C) Wild type DBD A-Cgt-T (red) versus A-Cgt-C (black). (D) Wild type DBD G-Cgt-C (blue) versus A-Cgt-C (black) (E) A477T DBD dimer mutant apo (grey) versus A477T DBD dimer mutant:A-Cgt-T (green) (F) A477T DBD dimer mutant A-Cgt-T (green) versus A-Cgt-C (orange).

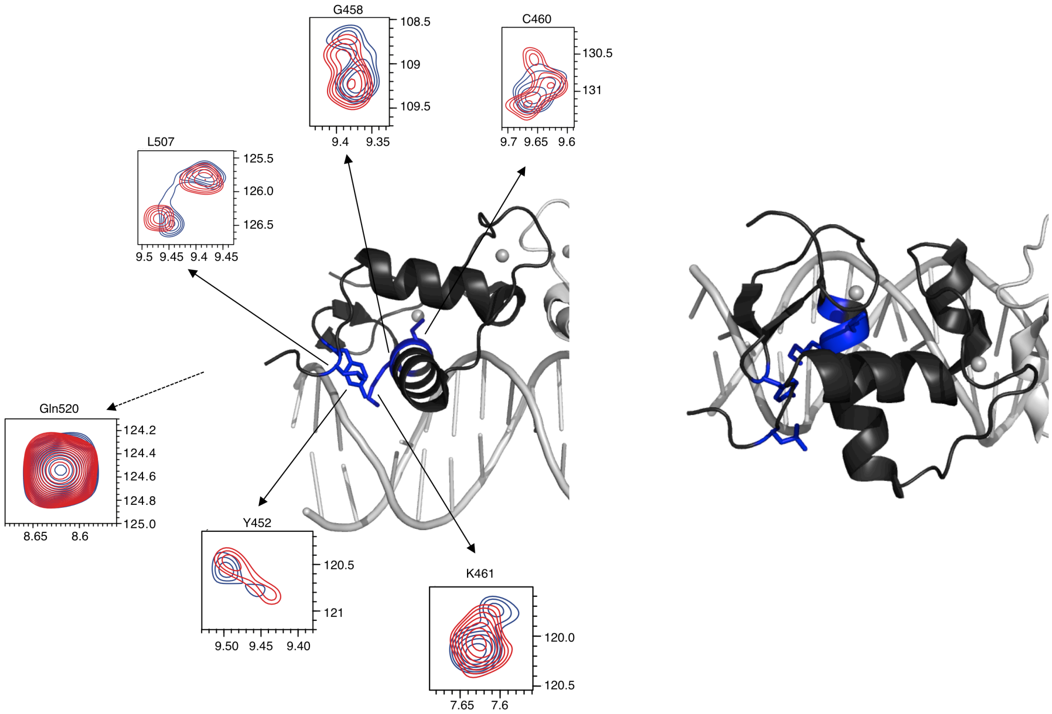

Supplementary Figure 6. ^{15}N-HSQC close-ups for selected peaks. ^{15}N-HSQC spectra of selected peaks for residues that show peak-splitting and non-overlapping spectra when comparing GR DBD in complex with either A/T (red) or G/C (blue) Cgt sequences are shown as zoom-ins and are projected onto chain A of the GR DBD structure (PDB: 3G9J) in blue. Left: side view; Right: top view.

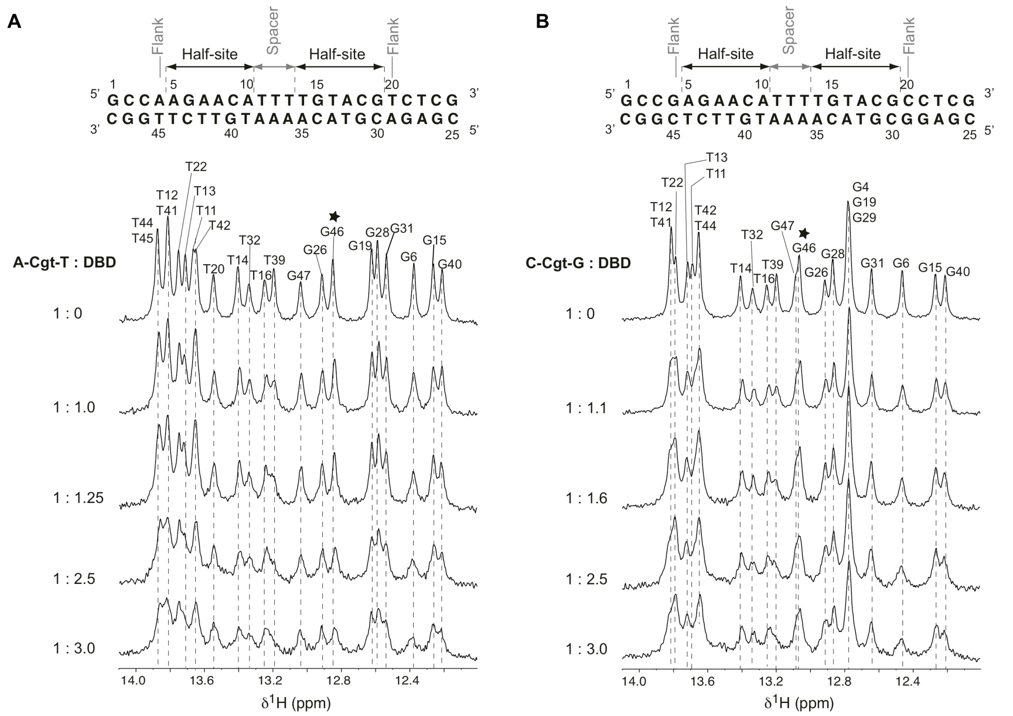

Supplementary Figure 7. GR DBD –DNA titration experiments. The imino proton regions of A/T-flanked (A) and G/C-flanked Cgt GBS (B) oligonucleotides are shown upon increasing the amount of protein. The secondary structure of both DNAs is indicated on the top. The assignments of imino proton spectra are based on the analysis of NOESY experiments. The DNA/protein ratios are indicated on the left. The resonance corresponding to the G46 imino proton, which exhibits a significant difference between the two oligonucleotides, is highlighted with a star.

A

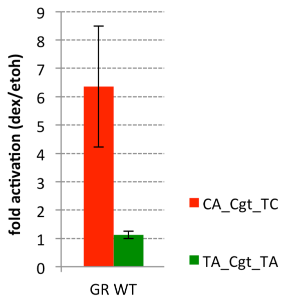

B

	CA-Cgt-TC	TA-Cgt-TA
K_D [μM]	0.41	0.47
S.E.M.	0.07	0.14

C

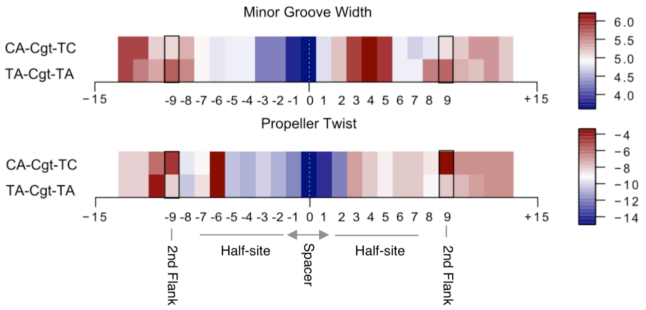

Supplementary Figure 8. Second flanking nucleotide also influences GR activity. (A) Comparison of transcriptional activation by the Cgt GBS sequence flanked by either CA/TC or TA/TA for wildtype GR. Average fold induction upon 1 μM dexamethasone (dex) treatment relative to ethanol vehicle (etoh) ± S.E.M. (n≥3) is shown. (B) Table of EMSA-derived DNA-binding constant (K_D) for second flanking site GBSs. Shown are averages and standard error of mean (S.E.M.) from 4 independent replicates. (C) Top: Predicted minor groove width and bottom: Predicted propeller twist for individual bases for the Cgt GBS flanked by either CA/TC or TA/TA.

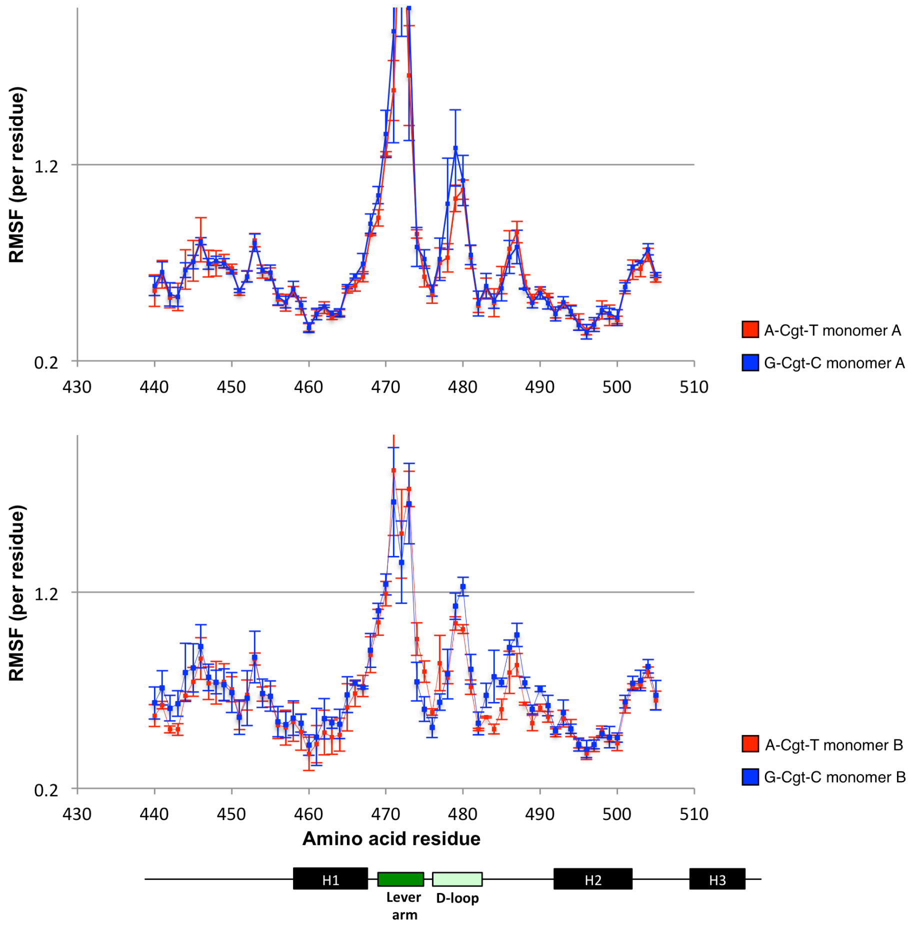

Supplementary Figure 9. Comparison of the flexibility of the DBD between A/T and G/C flanked sequences. RMSF of backbone atoms for each amino acid as indicated for (top) monomer A and (bottom) monomer B of GR over the first 100 ns MD simulation for GR bound to A/T flanked Cgt (red) and GR bound to G/C flanked Cgt (blue). Shown are averages and standard deviation from 3 different MD runs.

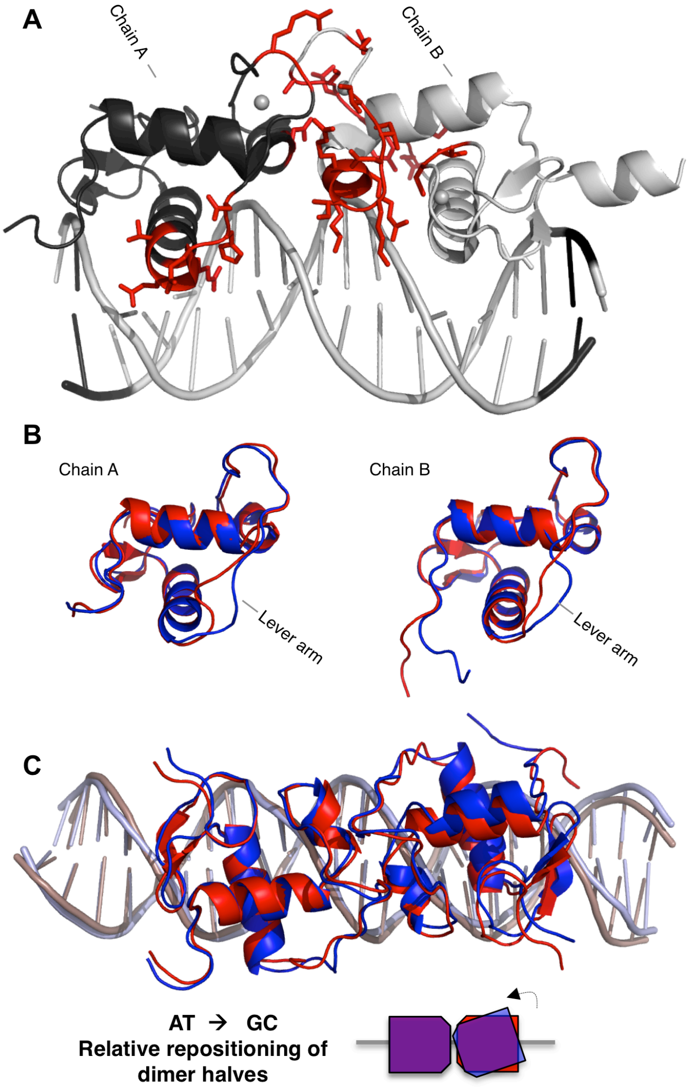

Supplementary Figure 10. MD simulations to probe the influence of flanking nucleotides on GR structure.

(A) GR DBD crystal structure with residues with RMSF differences >0.1 and those with non-overlapping standard deviations colored in red (comparing the first 100ns between MD simulations of DBD complexes with either A/T or G/C flanked Cgt). (B) Superposition of the median structures of GR DBD chain A (left) and chain B (right) for the last 50 ns of the MD simulations of the A/T (red) and G/C (blue) flanked Cgt complexes. (C) Comparison of GR backbone structure bound to A/T (red) and G/C (blue) flanked Cgt in MD simulations over last 50 ns aligned on chain A reveals relative repositioning of GR monomers.

Supplemental tables:

Supplementary Table 1. Oligos used for cloning GBS-reporters.

GBS:	Fw oligo	Rev oligo
G-FKBP5-C	CGAGAACAGGGTGTTCTCC	TCGAGGAGAACACCCTGTTCTCGGTAC
A-FKBP5-T	CAAGAACAGGGTGTTCTTC	TCGAGAAGAACACCCTGTTCTTGGTAC
G-Pal-C	CGAGAACAAAATGTTCTCC	TCGAGGAGAACATTTTGTTCTCGGTAC
A-Pal-T	CAAGAACAAAATGTTCTTC	TCGAGAAGAACATTTTGTTCTTGGTAC
G-Sgk-C	CGAGAACATTTTGTCCGCC	TCGAGGCGGACAAAATGTTCTCGGTAC
A-Sgk-T	CAAGAACATTTTGTCCGTC	TCGAGACGGACAAAATGTTCTTGGTAC
G-Cgt-C	CGAGAACATTTTGTACGCC	TCGAGGCGTACAAAATGTTCTCGGTAC
A-Cgt-T	CAAGAACATTTTGTACGTC	TCGAGACGTACAAAATGTTCTTGGTAC
G-Sgk-T	CGAGAACATTTTGTCCGTC	TCGAGACGGACAAAATGTTCTCGGTAC
A-Sgk-C	CAAGAACATTTTGTCCGCC	TCGAGGCGGACAAAATGTTCTTGGTAC
A-Cgt-C	CAAGAACATTTTGTACGCC	TCGAGGCGTACAAAATGTTCTTGGTAC
G-Cgt-T	CGAGAACATTTTGTACGTC	TCGAGACGTACAAAATGTTCTCGGTAC
A-FKBP5-2-T	CAAGAACATCCTGTGCCTC	TCGAGAGGCACAGGATGTTCTTGGTAC
G-FKBP5-2-C	CGAGAACATCCTGTGCCCC	TCGAGGGGCACAGGATGTTCTCGGTAC

Supplementary Table 2. Oligos used for site-directed-mutagenesis.

Construct:		
GR K465A	fw	AGCTGCAAAGTATTCTTTGCAAGAGCAGTGGAAGGAC
	rev	GTCCTTCCACTGCTCTTGCAAAGAATACTTTGCAGCT
TA-Sgk-TA	fw	CTCTATCGATAGGTACTAAGAACATTTTGTCCGTATCGAGATCTGCGATCTGCATC
	rev	GATGCAGATCGCAGATCTCGATACGGACAAAATGTTCTTAGTACCTATCGATAGAG
TA-Cgt-TA	fw	CTCTATCGATAGGTACTAAGAACATTTTGTACGTATCGAGATCTGCGATCTGCATC
	rev	GATGCAGATCGCAGATCTCGATACGTACAAAATGTTCTTAGTACCTATCGATAGAG
GR K511A	fw	GAACCTTGAAGCTCGAGCAACAAAGAAAAAAATC
	rev	GATTTTTTTCTTTGTTGCTCGAGCTTCAAGGTTC

Supplementary Table 3. Oligos used for EMSAs.

GBS:	Fw oligo:	Rev oligo:
G-Sgk-C	Cy5-ACCGAGAACATTTTGTCCGCCTC	GAGGCGGACAAAATGTTCTCGGT
A-Sgk-T	Cy5-ACCAAGAACATTTTGTCCGTCTC	GAGACGGACAAAATGTTCTTGGT
A-Cgt-T	Cy5-ACCAAGAACATTTTGTACGTCTC	GAGACGTACAAAATGTTCTTGGT
G-Cgt-C	Cy5-ACCGAGAACATTTTGTACGCCTC	GAGGCGTACAAAATGTTCTCGGT
CA-Cgt-TC	Cy5-TACCAAGAACATTTTGTACGTCTCG	CGAGACGTACAAAATGTTCTTGGTA
TA-Cgt-TA	Cy5-TACTAAGAACATTTTGTACGTATCG	CGATACGTACAAAATGTTCTTAGTA

Supplementary Table 4. primers used for qPCR.

Locus/Gene:	Fw primer	Rev primer
hRPL19 (ChIP & cDNA)	ATGTATCACAGCCTGTACCTG	TTCTTGGTCTCTTCCTCCTTG
pgl3promoter luc (ChIP)	GATGCGGTGGGCTCTATG	GAGTTAGGGGCGGGACTATG
hFKBP5 (ChIP)	GCATGGTTTAGGGGTTCTTG	TAACCACATCAAGCGAGCTG
TSC22D3 (cDNA)	TTA CAC CGC AGA ACC ACC AG	AGA TCG AAC AGG CCA TGG AT
Luc #2 (cDNA)	GCAGGTGTCGCAGGTCTT	GCGACGTAATCCACGATCTCTTTT

Supplementary Table 5. Oligos used for NMR experiments.

GBS:	Fw oligo:	Rev oligo:
Cgt, A-T	GCCAAGAACATTTTGTACGTCTCG	CGAGACGTACAAAATGTTCTTGGC
Cgt, G-C	GCCGAGAACATTTTGTACGCCTCG	CGAGGCGTACAAAATGTTCTCGGC
Cgt, A-C	GCCAAGAACATTTTGTACGCCTCG	CGAGGCGTACAAAATGTTCTTGGC
Cgt, G-T	GCCGAGAACATTTTGTACGTCTCG	CGAGACGTACAAAATGTTCTCGGC
